# Pituitary Carcinoma Revisited: A Systematic Review and Individual Patient Data Analysis of Clinicopathological Features, Treatment, and Survival

**DOI:** 10.3390/life16101602

**Published:** 2026-09-24

**Authors:** Bogdan Florin Iliescu, Loredana Mariana Curecheriu, Bogdan Costachescu, Gabriela Florenta Dumitrescu, Daniel Ilie Rotariu

**Affiliations:** 1 Department of Neurosurgery, Grigore T. Popa University of Medicine and Pharmacy Iasi, 11 Universitatii St., 700115 Iasi, Romaniadaniel-ilie.rotariu@umfiasi.ro (D.I.R.); 2 “N Oblu” Clinical Emergency Hospital Iasi, 2nd Ateneului St., 700309 Iasi, Romania

**Keywords:** pituitary carcinoma, pituitary neuroendocrine tumor, individual patient data, systematic review, survival analysis, temozolomide, immune checkpoint inhibitor, overall survival

## Abstract

Background: Pituitary carcinoma (PC) is a rare and aggressive pituitary neuroendocrine tumor (PitNET) defined by non-contiguous craniospinal or systemic metastatic spread. Evidence guiding treatment is limited to case reports and case series. This systematic review aimed to characterize the clinicopathological features, treatment patterns, and prognostic determinants of PC through pooled individual patient data (IPD) analysis. Methods: A systematic search of PubMed/MEDLINE and Embase (January 1990 to March 2026) was conducted in accordance with PRISMA 2020 guidelines (PROSPERO: CRD420261402665). Individual patient data were extracted for 56 pre-specified variables from the identified literature. Two additional institutional cases were analyzed separately as illustrative clinical examples and are not part of the pooled statistical cohort. Survival was estimated using the Kaplan–Meier method with log-rank tests. Univariate and multivariable Cox proportional hazards regression and binary logistic regression (outcome: long-survivor status, defined as OS > 24 months) were performed on the pooled literature cohort. Results: A total of 111 patients from 93 papers were included (51 males (45.9%); median age at PC diagnosis 54 years (IQR 40–61)), with corticotroph lineage predominating (47.7%). Median OS from PC diagnosis was 19 months (IQR 9.5–46); one-year OS was 67.7% and two-year OS 52.0%. Multimodal treatment was associated with significantly longer OS (median 45 vs. 12 months; log-rank *p* = 0.004). In multivariable Cox regression (n = 88; C-index 0.725), any objective treatment response (HR 0.245, 95% CI 0.101–0.593, *p* = 0.002), functional tumor status (HR 2.041, 95% CI 1.017–4.095, *p* = 0.045), and age at PC diagnosis (HR 1.018/year, *p* = 0.049) were independently associated with OS. Logistic regression identified multimodal treatment (OR 5.29, *p* = 0.009), any treatment response (OR 5.47, *p* = 0.006), and early metastasis (OR 0.18, *p* = 0.024) as independent predictors of long-survivor status. Ki-67 increased significantly from initial to PC-stage diagnosis (median 7% vs. 12%; Wilcoxon *p* < 0.001), which is, to our knowledge, the first demonstration of this longitudinal rise in a pooled pituitary carcinoma cohort. Conclusions: PC carries a poor prognosis, with a median OS of 19 months. Multimodal therapy and achievement of objective response are the strongest determinants of prolonged survival. Prospective registries with standardized molecular profiling are needed to advance precision management of this ultra-rare malignancy.

## 1. Introduction

Pituitary carcinomas (PCs) are rare tumors that represent approximately 0.1 to 0.2% of pituitary adenomas [1]. Their aggressive behavior is represented by malignant biology and craniospinal dissemination and/or systemic metastasis [2]. Early diagnosis of this pathology is difficult because PCs’ clinical manifestations relate to the primary lesion and its location [3,4].

This distinction is also relevant to the two institutional cases presented below. In Case 1, the lesion was initially managed as a prolactin-secreting pituitary adenoma and only later fulfilled the definition of PC when a non-contiguous posterior fossa lesion was histologically shown to be identical to the primary tumor. In Case 2, a previously treated ACTH-secreting pituitary adenoma similarly developed non-contiguous intracranial lesions, with biopsy confirmation of metastatic PitNET tissue establishing the diagnosis of PC. These trajectories illustrate why the terms “initial pituitary tumor” and “primary tumor” are used where appropriate and why PC should be regarded as a clinical-pathological diagnosis based on metastatic behavior rather than as a distinct morphology at initial presentation.

In the last WHO Classification paper published in 2022 the term metastatic pituitary neuroendocrine tumor (PitNET) was introduced for pituitary carcinoma. The change involves the actual problem of the tumor’s course [5]. There are no histological characteristics that indicate the metastatic potential at initial presentation. Only the clinical course and close follow-up reveal the final diagnosis. Current data and knowledge about the disease are built up by case presentations and single-center experiences.

This systematic review summarizes all the published cases of reported pituitary carcinomas and adds to two personal cases from our center, with a statistical analysis.

## 2. Materials and Methods

### 2.1. Study Design and Registration

This work comprises two complementary components. The first is a systematic review and pooled individual patient data (IPD) analysis, conducted in accordance with the Preferred Reporting Items for Systematic Reviews and Meta-Analyses (PRISMA) 2020 guidelines. The review protocol was prospectively registered with the International Prospective Register of Systematic Reviews (PROSPERO CRD420261402665) before the commencement of the formal database search. The second component consists of two original cases of histologically confirmed PC managed at the Department of Neurosurgery, University of Medicine and Pharmacy “Grigore T. Popa”, Iași, Romania, presented as illustrative cases in parallel to the pooled dataset and discussed separately in the context of the synthesized evidence.

### 2.2. Eligibility Criteria

#### 2.2.1. Inclusion Criteria

Studies were eligible for inclusion if they met all the following criteria:

1. Histological confirmation of diagnosis: pituitary carcinoma defined per current WHO and European Society of Endocrinology (ESE) criteria as a pituitary neuroendocrine tumor (PitNET) with demonstrated non-contiguous craniospinal or systemic metastases, with histological proof at the primary or metastatic site.
2. Extractable individual patient-level data: the minimum required variables were age at diagnosis, sex, tumor lineage, at least one treatment modality received, and follow-up duration or survival status.
3. Study design: case reports, case series, and retrospective cohort or registry-based studies.
4. Population: adult and pediatric patients, with no restriction on functional status, tumor lineage, or geographic region.
5. Publication period: January 1990 to the date of final search to ensure adequate histological and imaging documentation consistent with contemporary diagnostic standards.
6. Language: English-language publications only.

#### 2.2.2. Exclusion Criteria

Studies were excluded based on the following criteria:

1. Pituitary metastases originating from a non-pituitary primary malignancy.
2. Aggressive pituitary tumors (APTs) without confirmed non-contiguous metastasis, unless a carcinoma subgroup was separately identifiable within the same publication.
3. Abstracts, conference proceedings, editorials, and letters without extractable patient-level data.
4. Cases with absent histological confirmation or with insufficient clinical data to extract the minimum required variables.
5. Duplicate case reports: where the same patient was identified across multiple publications, the most complete and most recently updated report was retained, and the earlier reports were excluded.
6. Systematic reviews, narrative reviews, and meta-analyses that did not present new, extractable individual patient-level data; such reviews were retained and cited for contextual and background purposes but did not contribute to the pooled dataset.

### 2.3. Information Sources and Search Strategy

A systematic search was conducted in two electronic bibliographic databases: MEDLINE (via PubMed) and Embase. The search included papers with no start date, until the 30th of March 2026.

The PubMed search string, combining Medical Subject Headings (MeSH) and free-text terms, was as follows:

(“pituitary neoplasms”[MeSH Terms] OR “pituitary carcinoma”[tiab]
   OR “pituitary carcinomas”[tiab] OR “malignant pituitary”[tiab]
   OR “metastatic pituitary tumor”[tiab] OR “PitNET carcinoma”[tiab])
AND
(“neoplasm metastasis”[MeSH Terms] OR “metastas*”[tiab]
   OR “craniospinal dissemination”[tiab] OR “spinal metastasis”[tiab])
AND
(“treatment”[tiab] OR “management”[tiab] OR “surgery”[tiab]
   OR “radiotherapy”[tiab] OR “temozolomide”[tiab]
   OR “immunotherapy”[tiab] OR “checkpoint inhibitor”[tiab]
   OR “survival”[tiab] OR “outcome”[tiab])

An equivalent search strategy, employing Emtree controlled vocabulary and free-text terms, was applied in Embase.

### 2.4. Study Selection

All retrieved records were exported to the reference management platform Rayyan (Rayyan Systems Inc., Cambridge, MA, USA) and automatically deduplicated, with manual verification of any residual duplicates. Title and abstract screening were performed independently by two reviewers, applying the pre-specified eligibility criteria. Disagreements were resolved by discussion. Unresolved conflicts were adjudicated by a third reviewer. Full-text retrieval and assessment were conducted for all records not excluded at the abstract stage. The screening and selection process is summarized in a PRISMA 2020 flow diagram (Figure 1).

### 2.5. Data Extraction

Data was extracted independently by two reviewers (LMA and BFI) using a pre-specified, pilot-tested extraction template developed in Microsoft Excel 2021 (Microsoft Corporation, Redmond, WA, USA). Disagreements were resolved by discussion and, where consensus could not be reached, by arbitration from a third reviewer (DR).

The following individual patient-level variables were extracted:

(1) Baseline characteristics:
  - Demographics: age at initial diagnosis (years), sex, country of report, year of publication.
  - Tumor characteristics: lineage per 2022 WHO PitNET classification (corticotroph, lactotroph, somatotroph, thyrotroph, gonadotroph, null cell, plurihormonal, or not otherwise specified); functional status and hormone type; maximum tumor diameter (mm); invasiveness per Hardy–Wilson classification [6,7] or equivalent.
  - Pathological markers: Ki-67 proliferation index (%); mitotic count per 10 high-power fields; p53 immunopositivity; Crooke’s cell morphology (corticotroph tumors).
  - Molecular markers: MGMT expression by immunohistochemistry and/or promoter methylation status; TP53 mutational status; ATRX expression; SF3B1 variant; mismatch repair status (MSI/MMR); tumor mutational burden (mut/Mb); PD-L1 expression (%).
  - Treatment at initial diagnosis: surgical approach (transsphenoidal or transcranial), extent of resection, adjuvant radiotherapy, and medical therapy (dopamine agonist, somatostatin analogue, or other).
(2) Pituitary carcinoma diagnosis:
  - Time from initial pituitary tumor diagnosis to PC diagnosis (months); number of surgical interventions before PC diagnosis.
  - Ki-67 index of metastatic lesion; histological lineage of metastasis, where reported.
  - Site(s) of metastasis: craniospinal (intradural, leptomeningeal, vertebral) versus systemic (liver, lymph nodes, bone, or other); number of distinct metastatic sites; histological confirmation at the metastatic site (yes/no).
(3) Treatment after PC diagnosis (in sequence):
  - Surgical resection (primary or metastatic site); radiotherapy type (conventional fractionated external beam radiotherapy, stereotactic radiosurgery, proton beam, craniospinal irradiation, or whole-brain radiotherapy) and total dose (Gy); temozolomide (schedule and number of cycles); immune checkpoint inhibitors (agent and number of cycles); bevacizumab; peptide receptor radionuclide therapy (PRRT); and any other systemic therapy.
  - Best radiological response to each modality (complete response (CR), partial response (PR), stable disease (SD), or progressive disease (PD), per RECIST [8] or equivalent criteria where reported).
  - Biochemical response for functioning tumors (CR, PR, SD, PD, or not applicable); treatment-related adverse events of grade ≥ 3, graded according to the Common Terminology Criteria for Adverse Events (CTCAE) [9] when a formal grading system was reported in the source publication. For publications predating CTCAE or reporting no formal grading instrument, events explicitly described as severe or life-threatening, or events requiring treatment discontinuation or hospitalization, were coded as grade ≥ 3.
(4) Outcomes:
  - Overall survival from PC diagnosis (months); vital status at last follow-up (alive or dead); cause of death.

### 2.6. Risk of Bias Assessment

Given that the eligible evidence base consists exclusively of case reports and small case series, formal comparative risk of bias tools were not applicable. Reporting completeness was therefore assessed using the JBI Critical Appraisal Checklist for Case Reports (Joanna Briggs Institute, 2020) [10], an eight-item instrument developed specifically for single-patient reports and, unlike comparator-dependent tools such as the Newcastle–Ottawa Scale or ROBINS-I, appropriate for a literature base composed exclusively of case reports and small case series lacking a control arm. The checklist evaluates patient demographic clarity, history, clinical presentation, diagnostic workup, treatment description, post-intervention outcome, adverse event reporting, and clinical takeaway. Items were scored Yes (1) or No/Unclear (0); total scores (0–8) were categorized as low (4–5), moderate (3), or high concern (≤2). Assessment was performed independently by two reviewers (LMA and BFI); disagreements were resolved by consensus. A pre-specified sensitivity analysis restricted outcomes to cases with low concern (score ≥ 4).

### 2.7. Statistical Analysis

Given the rarity of PC and expected clinical heterogeneity, formal meta-analytic pooling of aggregate effect estimates was not pre-planned; analysis was based on pooled IPD following PRISMA-IPD extension guidelines. Consistent with PRISMA-IPD methodology, the pooled statistical cohort (n = 111) comprises exclusively patients identified through the systematic search, screening, and eligibility process summarized in Figure 1. The two institutional cases, being unpublished and not ascertained through this systematic pipeline, were therefore not incorporated into the pooled IPD analysis to avoid introducing non-systematically identified data into a systematically derived dataset; they are instead reported separately (Section 3.7) as illustrative clinical vignettes, contextualized against the pooled findings in the Discussion. 

Overall survival from PC diagnosis (time from confirmed non-contiguous metastasis to death or censoring) was estimated using the Kaplan–Meier method; subgroup comparisons used the log-rank test. Univariate Cox proportional hazards regression was performed for eight candidate covariates (age, sex, corticotroph lineage, functional status, multimodal treatment, early metastasis, any response, number of metastatic sites); covariates with *p* < 0.20 entered a multivariable Cox model. Independent predictors of long-survivor status (OS > 24 months) were identified by binary logistic regression. Fisher’s exact test with OR and 95% CI was used for pre-specified 2 × 2 associations. The Wilcoxon signed-rank test compared paired Ki-67 values. All analyses were performed using JASP version 0.19 (JASP Team, University of Amsterdam, the Netherlands) and Python 3.12 (scipy 1.x). Two-tailed *p* < 0.05 was considered statistically significant.

### 2.8. Institutional Cases

Two cases managed at the Department of Neurosurgery, University of Medicine and Pharmacy “Grigore T. Popa”, Iași, Romania were included using an identical extraction template. Written informed consent was obtained from the patient or their legal representatives. All data were handled in accordance with the Declaration of Helsinki. The cases are presented separately in parallel with the pooled cohort and compared against the synthesized evidence in the Discussion section.

## 3. Results

### 3.1. Study Selection and Patient Characteristics

A total of 111 patients with histologically confirmed pituitary carcinoma were identified from publications spanning 1991 to 2026 and included in the IPD analysis. The PRISMA flow diagram is shown in Figure 1. Patient characteristics are summarized in Table 1 [11,12,13,14,15,16,17,18,19,20,21,22,23,24,25,26,27,28,29,30,31,32,33,34,35,36,37,38,39,40,41,42,43,44,45,46,47,48,49,50,51,52,53,54,55,56,57,58,59,60,61,62,63,64,65,66,67,68,69,70,71,72,73,74,75,76,77,78,79,80,81,82,83,84,85,86,87,88,89,90,91,92,93,94,95,96,97,98,99,100,101,102,103,104,105,106,107,108,109,110,111,112,113,114,115,116,117,118,119,120,121].

The cohort comprised 51 males (45.9%) and 60 females (54.1%). Corticotroph lineage predominated (n = 53, 47.7%), followed by lactotroph (n = 33, 29.7%), null cell (n = 13, 11.7%), somatotroph (n = 6, 5.4%), and other lineages (n = 6, 5.4%). Median age at initial pituitary tumor diagnosis was 47 years (IQR 34–55; range 9–75) and at PC diagnosis it was 54 years (IQR 40–61). Median latency from initial diagnosis to PC was 72 months (IQR 43–132; range 0–516 months), reflecting the characteristically protracted clinical course of this malignancy. Craniospinal metastases were identified in 68 patients (61.3%) and systemic metastases in 62 (55.9%). Nineteen patients (17.1%) developed metastases within 24 months of their initial diagnosis (early metastasis group).

### 3.2. Overall Survival and Kaplan–Meier Analysis

OS data were available for 99 of 111 patients (89.2%). Median OS from PC diagnosis was 19 months (IQR 9.5–46; range 0–252 months). The estimated one-year OS was 67.7% and two-year OS was 52.0%. A total of 58 patients (53.7%) died during the observation period; 50 (46.3%) were alive or censored at last follow-up.

Kaplan–Meier survival curves with log-rank tests were generated for six pre-specified subgroups (Table 2). Multimodal treatment was associated with significantly longer OS compared to single-modality therapy (median 45 vs. 12 months; log-rank χ^2^ = 8.337, df = 1, *p* = 0.004). Achievement of any objective response was the strongest predictor on KM analysis (median OS not reached vs. 21 months; log-rank *p* < 0.001). A significant difference in OS was observed across Ki-67 categories at initial diagnosis (log-rank χ^2^ = 7.574, df = 2, *p* = 0.023; n = 45 with available data): median OS was 156, 32, and 12 months for Ki-67 < 3%, 3–9%, and ≥10%, respectively. Corticotroph lineage was not significantly associated with OS (median 45 vs. 24 months; *p* = 0.330).

### 3.3. Treatment and Response

Treatment data were available for all 111 patients (Table 3). Temozolomide achieved an objective response rate (ORR; CR + PR) of 35% among evaluable patients (14/40). Immune checkpoint inhibitors (ICIs; predominantly pembrolizumab) demonstrated an ORR of 67% (4/6 evaluable), although the sample was small (n = 8 treated). Most patients (n = 73, 65.8%) received multimodal treatment (≥2 modalities).

### 3.4. Cox Proportional Hazards Regression

Univariate and multivariable Cox regression results are presented in Table 4. Of eight pre-specified covariates, four entered the multivariable model (univariate *p* < 0.20): age at PC diagnosis, functional status, multimodal treatment, and any treatment response. In the multivariable model (n = 88, events = 54; C-index = 0.725; likelihood ratio χ^2^ = 25.874, *p* < 0.001), any objective response was the strongest independent predictor of reduced mortality hazard (HR 0.245, 95% CI 0.101–0.593, *p* = 0.002). Functional tumor status was associated with increased hazard (HR 2.041, 95% CI 1.017–4.095, *p* = 0.045). Age at PC diagnosis retained borderline significance (HR 1.018/year, 95% CI 1.000–1.036, *p* = 0.049). Multimodal treatment did not retain independent significance (HR 0.678, *p* = 0.189), likely reflecting collinearity with response status.

### 3.5. Predictors of Long-Survivor Status

Binary logistic regression (outcome: OS > 24 months; n = 87 complete cases) identified three independent predictors of long-survivor status (Nagelkerke R^2^ = 0.390; AUC = 0.818; Table 5). Multimodal treatment (OR 5.29, 95% CI 1.42–19.7, *p* = 0.009) and any treatment response (OR 5.47, 95% CI 1.62–18.5, *p* = 0.006) were independently associated with significantly higher odds of long-term survival. Early metastasis was independently associated with reduced odds of long-survivor status (OR 0.18, 95% CI 0.04–0.81, *p* = 0.024). Age at PC diagnosis showed a borderline inverse trend (OR 0.967/year, *p* = 0.060). No influential cases were identified (Cook’s distance).

### 3.6. Additional Analyses

Ki-67 increased significantly from initial pituitary tumor diagnosis to PC-stage diagnosis in 38 paired cases (median 7% vs. 12%; Wilcoxon signed-rank *p* < 0.001), consistent with progressive malignant transformation (Table 6). Any treatment response was strongly associated with long-survivor status on Fisher exact analysis (OR 6.00, 95% CI 1.89–21.6, *p* < 0.001), as was multimodal treatment (OR 5.27, 95% CI 1.71–19.7, *p* = 0.002). No significant association was observed between TMZ response and corticotroph lineage (*p* = 0.327). Age at PC diagnosis was inversely correlated with OS (Spearman ρ = −0.229, *p* = 0.023). JBI appraisal (n = 66 cases completed) classified 30.3% as low concern, 63.6% moderate concern, and 6.1% high concern; ROB category was not significantly associated with OS (Kruskal–Wallis *p* = 0.576), supporting the robustness of survival estimates.

### 3.7. Personal Cases

Two additional patients with histologically confirmed pituitary carcinoma, managed in our department, are presented individually below to illustrate the clinical trajectory and are compared against the synthesized evidence in the Discussion section.

*Case 1*

We present a 50-year-old woman with a known prolactin-secreting pituitary adenoma, treated with dopamine agonists for 36 months. The patient presented emergently with intracranial hypertension, left oculomotor palsy, and bitemporal hemianopia. MRI demonstrated a 4.0 cm sellar and suprasellar lesion with Knosp grade 4 invasion of the left cavernous sinus and optic chiasm compression, consistent with hemorrhagic apoplexy (Figure 2A,B). Emergent left pterional craniotomy achieved subtotal resection, with residual tumor in the cavernous sinus (Figure 2C,D); dopamine agonist therapy was continued post-operatively, followed by Gamma Knife radiosurgery (18 Gy) to the residual tumor.

Four years later, the patient developed intracranial hypertension and left hemiparesis; MRI showed progression of the sellar lesion together with a non-contiguous posterior fossa lesion (Figure 3), which was resected via a far-lateral suboccipital approach. Histopathology confirmed a pituitary neoplasm morphologically and immunophenotypically identical to the primary tumor, establishing the diagnosis of pituitary carcinoma (Figure 4). Three months later, a new right cerebellopontine angle metastasis appeared (Figure 5); salvage Gamma Knife radiosurgery (20 Gy) was delivered to this lesion, and temozolomide (150 mg/m^2^/day × 5 days every 28 days) was initiated. After four cycles, imaging showed partial response of the cerebellopontine angle lesion but progression of the sellar and dural disease (Figure 3); temozolomide was discontinued for toxicity.

Second-line lomustine was stopped after a single cycle because of severe fatigue and declining performance status. The patient died 32 months after the diagnosis of pituitary carcinoma, approximately ten years after the initial adenoma diagnosis, following progressive multifocal intracranial disease (Figure 5 and Figure 6).

*Case 2*

We present the case of a 65-year-old woman with a history of ACTH-secreting pituitary adenoma, initially treated with transsphenoidal resection for pituitary apoplexy in 2015, followed by adjuvant Gamma Knife radiosurgery (18 Gy) in 2016 with initial hormonal normalization. Biochemical relapse and local recurrence in 2019 were managed with a second radiosurgery session (21 Gy). In 2020, the patient presented with poorly controlled hypertension and clinical features of active Cushing’s disease. Contrast-enhanced MRI demonstrated recurrent sellar tumor with clival and suprasellar extension, together with two non-contiguous lesions—one at the floor of the third ventricle and one in the cerebellar vermis (Figure 7). Stereotactic biopsy of the vermian lesion confirmed metastatic PitNET tissue, establishing the diagnosis of pituitary carcinoma (Figure 8). Temozolomide (150 mg/m^2^/day × 5 days every 28 days) was initiated but discontinued after three cycles because of clinical deterioration. The disease continued to progress, and the patient died six months after diagnostic confirmation, secondary to massive pulmonary thromboembolism.

## 4. Discussion

PCs are extremely rare tumors, occurring in approximately 0.1–0.2% of pituitary neoplasms initially diagnosed as pituitary adenomas [1]. The 2022 WHO classification defines PC by non-contiguous craniospinal and/or systemic dissemination of a PitNET [5].

Our systematic review provides a pooled individual patient data analysis of 111 cases published over the last three decades. Therefore, this study provides one of the largest quantitative characterizations of PCs to date, with a median overall survival of 19 months from carcinoma diagnosis. Our search identified multimodal treatment, objective treatment response, functional status and age at diagnosis as independent prognostic determinants.

These findings are generally consistent with the two European Society of Endocrinology (ESE) surveys, which remain the principal comparator cohorts in this field [120]. The surveys primarily addressed aggressive pituitary tumors and included subgroup analyses of identified pituitary carcinomas, reporting poor long-term prognosis [32]. Our cohort’s median OS of 19 months sits within the range reported by the ESE surveys, reinforcing that survival estimates for PC have remained essentially unchanged despite two decades of incremental therapeutic additions and underscoring the need for the individual patient treatment plan.

The strong association between multimodal therapy and prolonged survival (median 45 vs. 12 months) is consistent with the treatment approach endorsed by the ESE clinical practice guidelines. The revised practical guideline recommends early coordinated multidisciplinary management, combining surgery, radiotherapy and systemic therapy rather than sequential single-modality escalation [121]. However, this association may be confounded, as patients who are fit enough to tolerate multiple treatment lines may have inherently less aggressive disease or better performance status.

Treatment response was associated with longer survival and, in our cohort, emerged as the single strongest independent predictor of survival (HR 0.245). It provides indirect support to the growing use of second-line immune checkpoint inhibitors in temozolomide-refractory disease [50,121,122]. This finding supports consideration of second-line immunotherapy after temozolomide failure, while acknowledging the absence of randomized evidence.

Of the analyzed population, 47.7% had a corticotroph lineage, consistent with reports of a greater representation of this lineage among aggressive PCs. The association between functional status and worse survival (HR 2.041) seems counterintuitive, but this pattern mirrors prior observations and raises the hypothesis that corticotroph tumors may represent a marker of aggressiveness and malignant potential [123,124].

A significant finding was the rise in Ki-67 index between initial diagnosis and PC diagnosis (7% vs. 12%, *p* < 0.001). This result provides direct longitudinal evidence of the progressive malignant transformation process that underlies the current classification [121]. Mitotic count, the other principal proliferation marker in pituitary tumor pathology, was reported far less consistently across the literature than Ki-67, with an extractable value at initial diagnosis in only 27 of 111 cases (24.3%; median 1 mitosis/10 HPF, IQR 0–3.5), and was not recorded at PC-stage diagnosis in any source report, precluding a paired analysis analogous to that performed for Ki-67. Where both markers were available (n = 19), mitotic count correlated significantly with Ki-67 index (Spearman ρ = 0.499, *p* = 0.030), supporting their shared biological basis as proliferation indices. This reporting gap itself is informative: it underscores the inconsistency of quantitative mitotic reporting in the published pituitary carcinoma literature and reinforces the case for standardized, mandatory reporting of both Ki-67 and mitotic count in future case descriptions and prospective registries.

This finding is consistent with the five-tiered clinicopathological classification proposed by Trouillas and colleagues, in which proliferative grade has been prospectively validated as an independent predictor of recurrence and progression [125]. Sequential biopsies published in case series also show a difference in Ki-67 measures at different time-points [49]. Taken together, these data support consideration of serial Ki-67 assessment during tumor surveillance, as a rising proliferative index may signal progression toward a metastatic phenotype.

The first presented case had survival longer than the pooled median (32 months from PC diagnosis), consistent with the multimodal treatment and objective-response associations identified in the pooled analysis. Notably, this patient’s initial adenoma was already Knosp grade 4 at presentation, years before malignant transformation, a feature consistent with aggressive pituitary tumor behavior.

The second case, a corticotroph-lineage tumor, survived only 6 months from PC diagnosis, substantially shorter than the pooled median. The patient did not achieve an objective response to temozolomide and was not escalated to a second-line systemic agent before clinical deterioration. Whether earlier immunotherapy would have altered this course cannot be determined from this case. This patient also had active Cushing’s disease at the time of PC diagnosis, offering a clinical illustration of the pooled finding that functioning tumor status is independently associated with worse survival.

Taken together, these two cases illustrate the central prognostic signal of the pooled analysis: survival in pituitary carcinoma appears to track closely with treatment response and clinical status at diagnosis.

## 5. Conclusions

This systematic review and individual patient data analysis of 111 cases identifies multimodal treatment, objective treatment response, functional tumor status and age at diagnosis as independent predictors of survival in pituitary carcinoma. The median OS of 19 months remains unchanged from prior ESE surveys despite treatment innovation.

Two unpublished institutional cases, analyzed separately, illustrated the same response–survival pattern observed in the pooled analysis, providing clinical context for the statistical findings. Given the near-absence of molecular data across the literature, prospective registries with standardized molecular profiling are needed to clarify which patients benefit most from second-line immunotherapy after temozolomide failure.

## Figures and Tables

**Figure 1:**
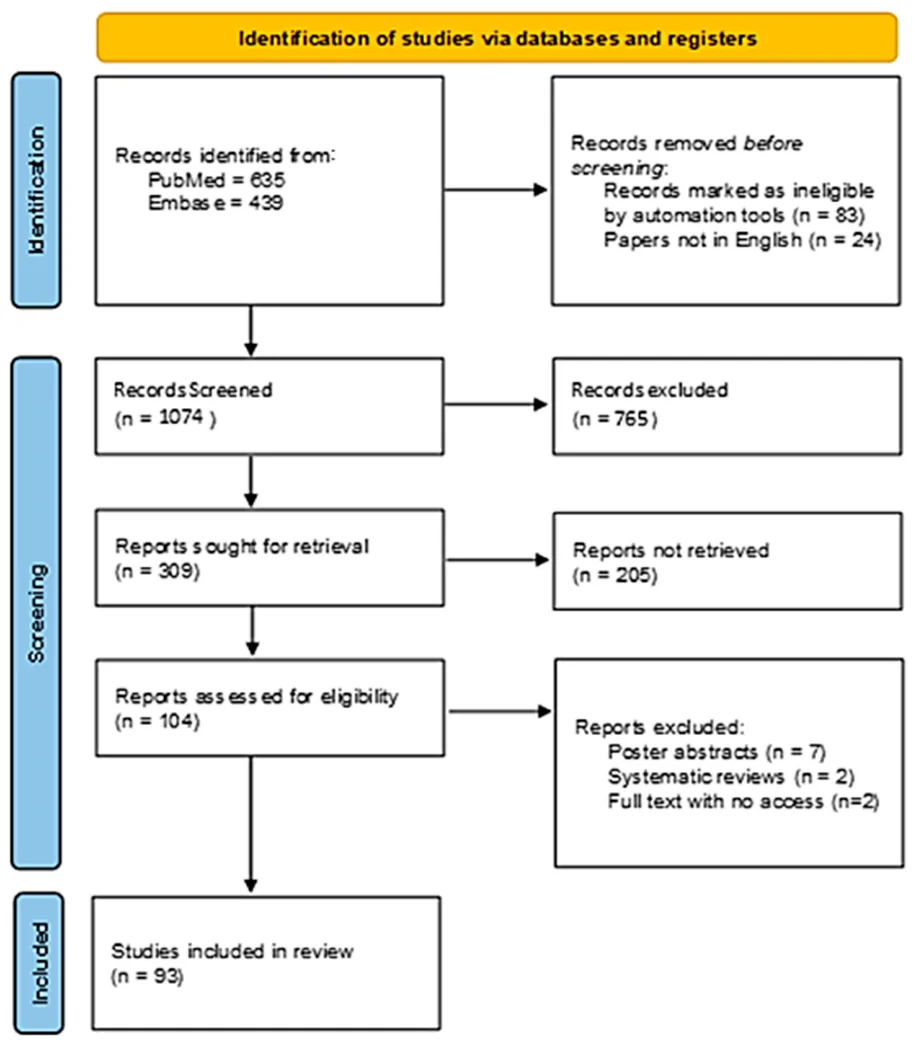
PRISMA diagram showing the search and final number of included studies.

**Figure 2:**
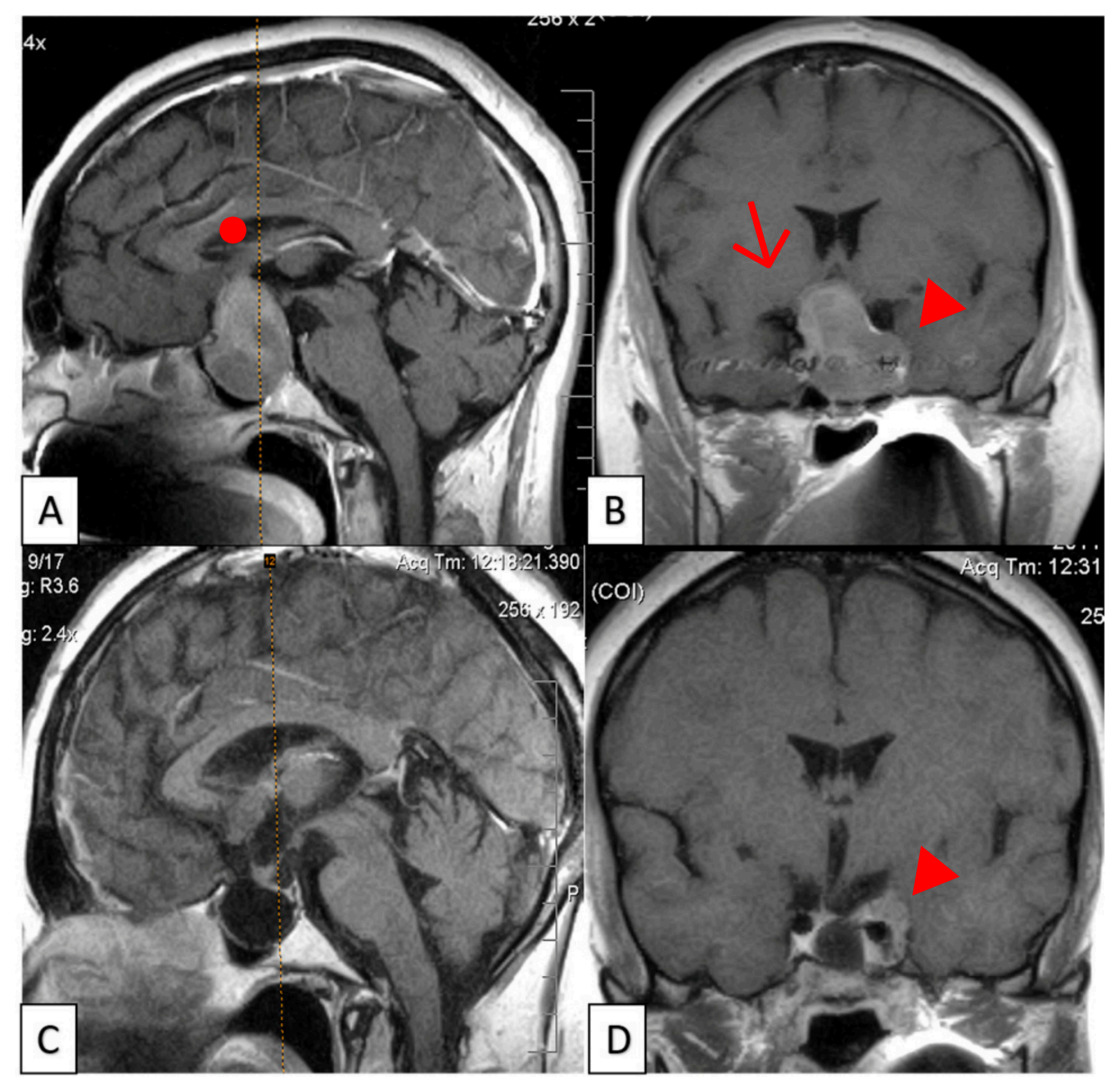
Pre-operative MRI images T1w + contrast enhancement showing a sellar lesion with a mixed structure suggestive of pituitary apoplexy (**A**), with compression of the optic chiasm and invasion of the left cavernous sinus (point); (**B**–**D**) post-operative MRI showing the decompression of the optic apparatus ((**C**) sagittal and (**D**) coronal sections) showing tumor remnant at the level of the left cavernous sinus (arrowhead).

**Figure 3:**
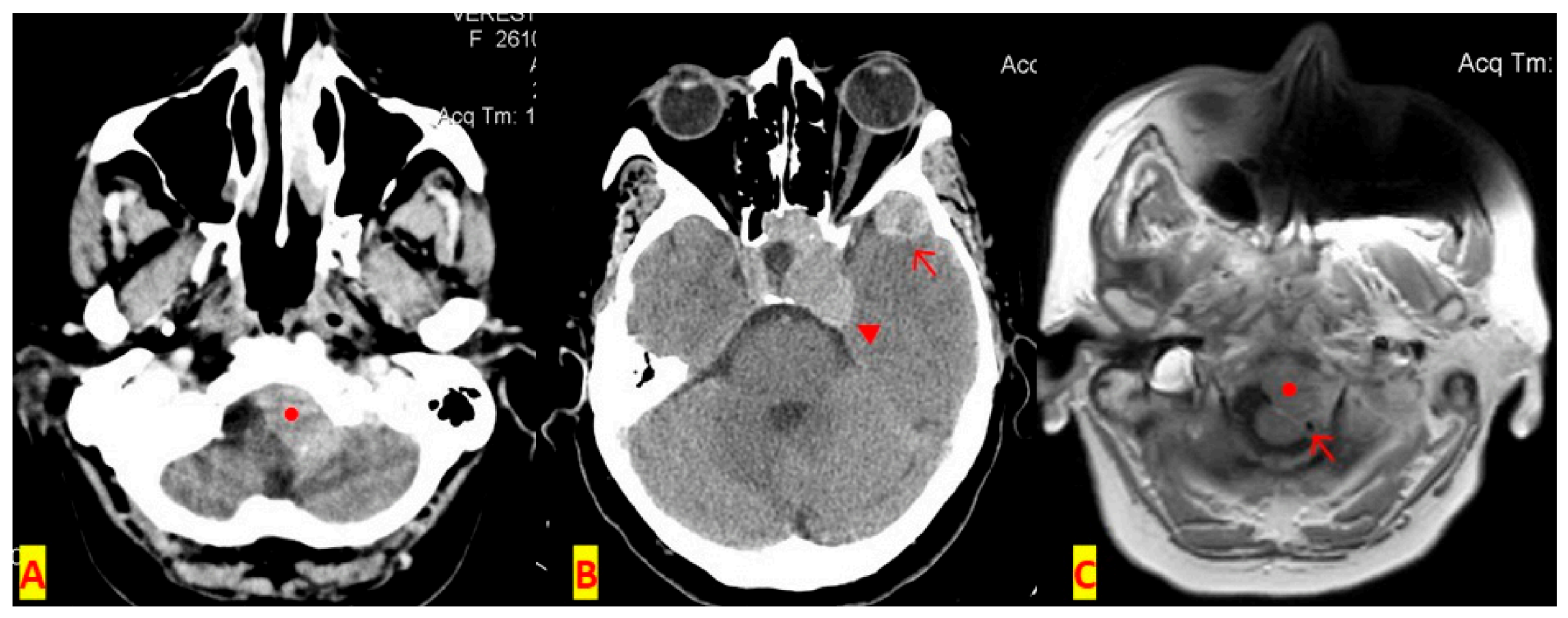
(**A**) CT scan with contrast enhancement showing a lesion of the posterior fossa (point). (**B**) axial section at the level of the sella showing the progression of the tumor remnant (arrowhead) and another lesion at the level of the temporal pole (arrow). (**C**) MRI T1w with contrast enhancement showing the lesion plated at the level of the skull base and the relationship with the left PICA (arrow).

**Figure 4:**
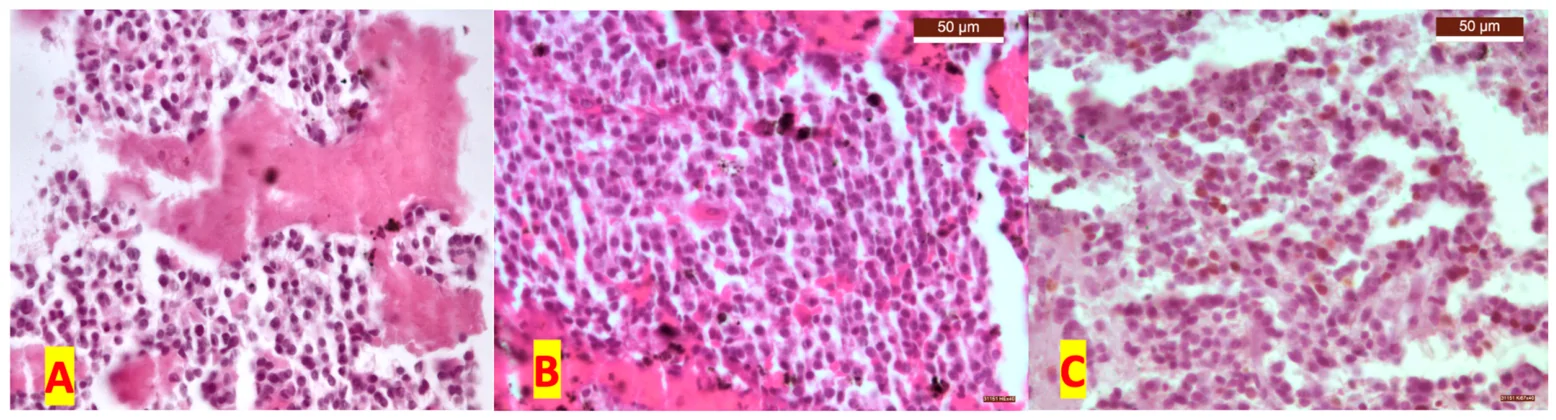
Histological images from the 1st case of pituitary carcinoma. (**A**) Images from the first surgery showing sheets of monotonous tumor cells, frequently arranged in perivascular pseudorosettes. Tumor cells were uniform, polygonal (epithelioid), featuring moderately abundant, chromophobic cytoplasm and round nuclei, with fine, punctate chromatin (“salt and pepper” appearance). Stroma was represented by a delicate and rich vascular network (HE staining, ×400). (**B**) Same case’s histological images of the posterior fossa lesion showing a tumor composed of discohesive pituitary cells arranged in nests separated by a fibrovascular stroma. The majority of tumor cells have an epithelioid appearance, with relatively pleomorphic nuclei, finely stippled (“salt and pepper”) chromatin, inconspicuous nucleoli, and moderately abundant chromophobic cytoplasm that is morphologically identical to the primary tumor (1st surgery) (HE, ×400). (**C**) Ki-67 labeling index of 16%, focally, suggesting an aggressive behavior (immunohistochemical staining, ×400).

**Figure 5:**
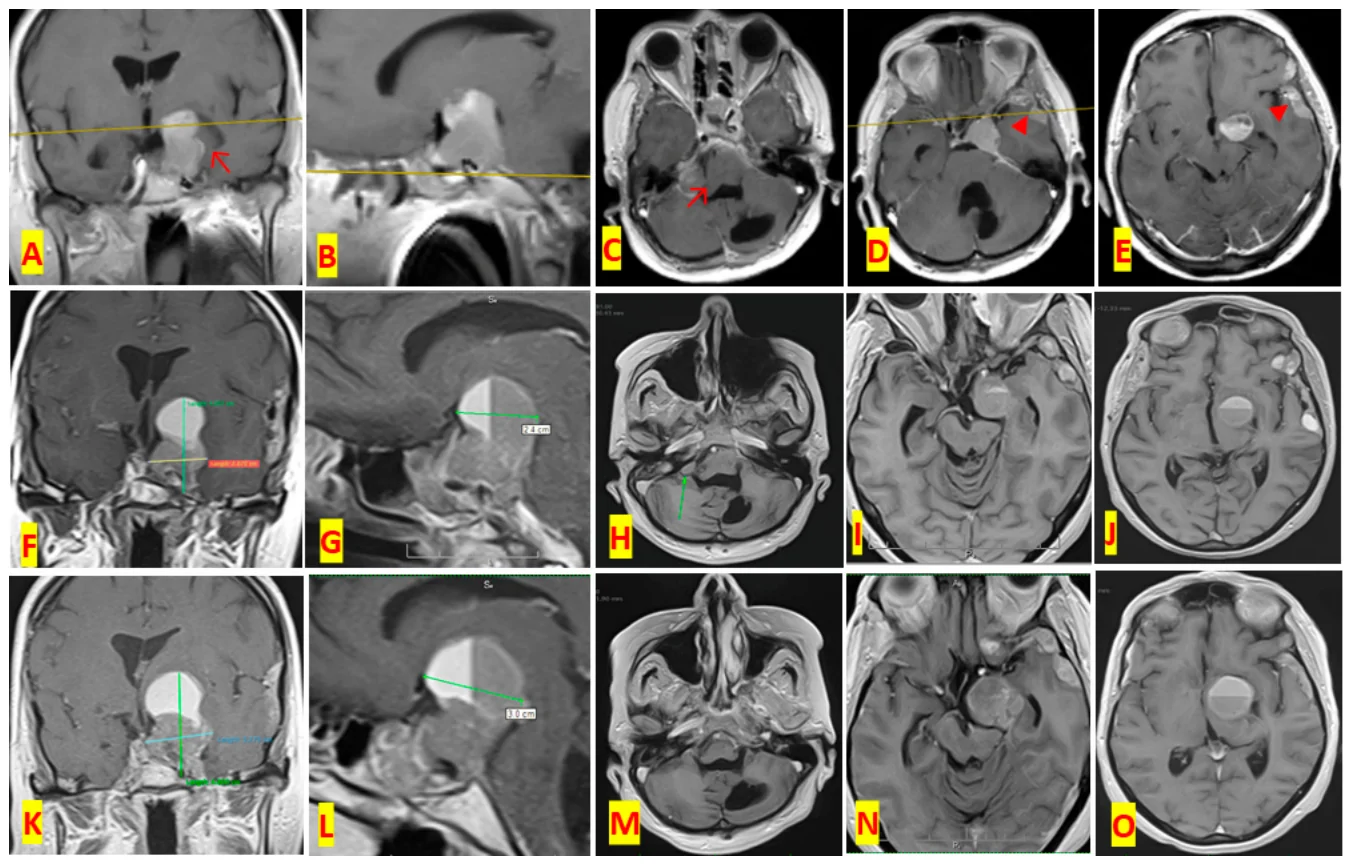
MRI images, same sections showing the evolution of the lesions over time. (**A**–**E**) MRI imaging at 3 months after the second surgery showing further progression of the lesions (**A**,**B**,**D**,**E**) with dissemination in the subdural space ((**D**,**E**) arrowheads) and the development of new ones (**C**) —new lesion at the level of the right cerebellopontine angle (arrow). (**F**–**J**) MRI imaging one year after the second surgery (6 months after RxT and temozolomide) showing the progression of the lesions and shrinkage of the CP angle lesion (**H**). (**K**–**O**) MRI imaging 2 years after the second surgery (18 months after RxT and temozolomide) showing further expansion of the parasellar lesion, with intratumoral necrosis, the progression of the temporal subdural and the regression of the subdural frontotemporal lesions but with complete disappearance of the right CP angle lesion (**H**).

**Figure 6:**
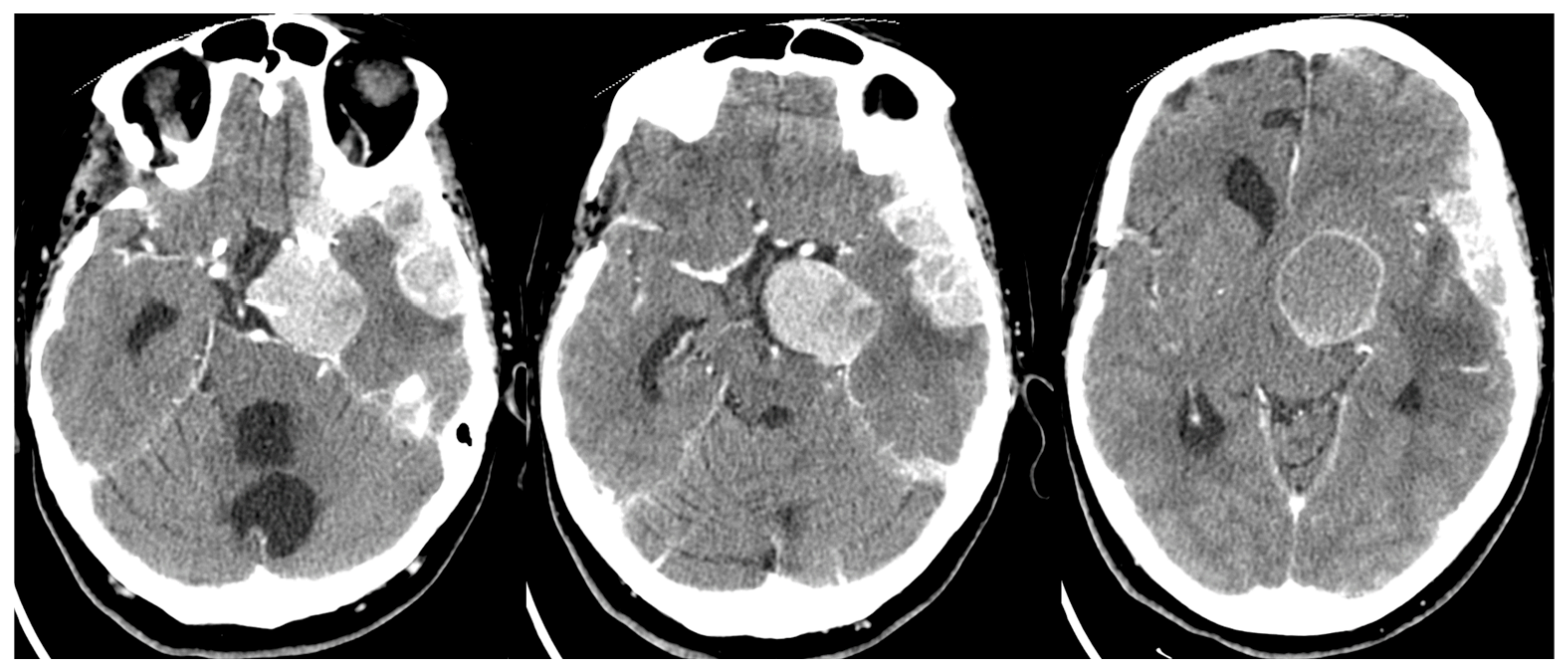
CT scan performed for the evaluation of newly occurring seizures (6 years post-op)—showing the progression of all previously described lesions, with perilesional edema and important mass effect and displacement of the ventricles and midline structures.

**Figure 7:**
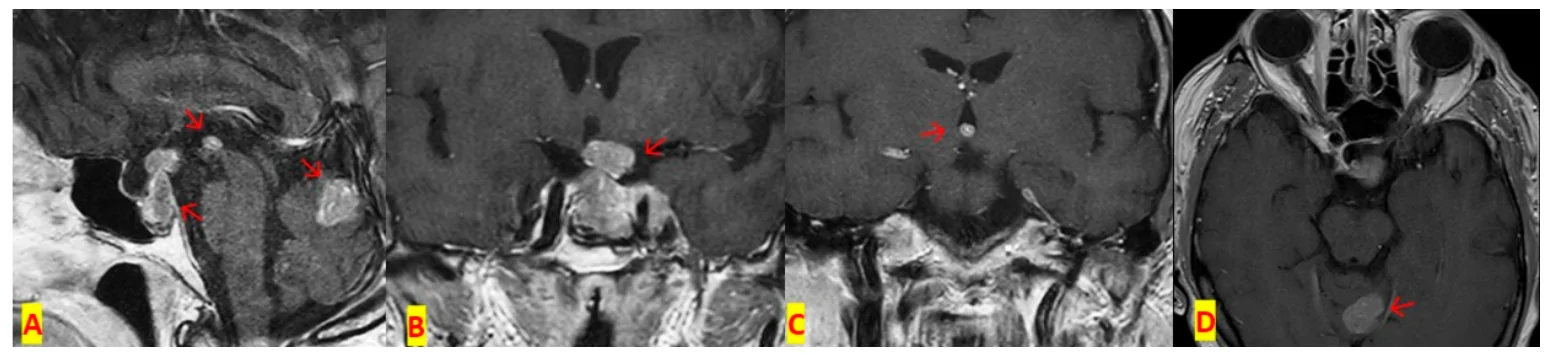
MRI images T1w with contrast enhancement, showing the lesion at the level of the sella with dissemination at the level of the 3rd ventricle and at the level of the cerebellar vermis in the posterior fossa. Arrows point to the lesions at all locations (**A**), sellar lesion (**B**), 3rd ventricle (**C**) and vermian lesion (**D**).

**Figure 8:**
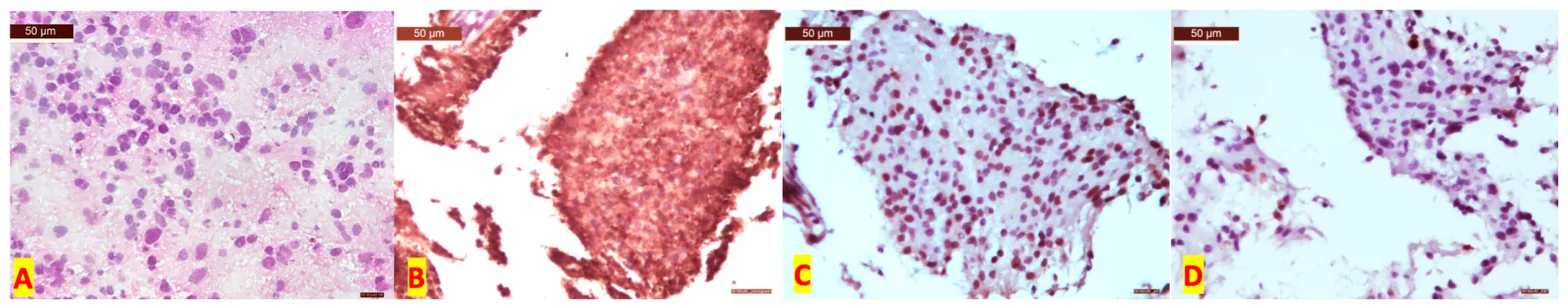
Histological images of the vermian lesion (biopsy) in the 2nd case of pituitary carcinoma showing sparsely cellular tumor composed of medium- to large-sized pituitary cells with indistinct cell borders, scant faintly eosinophilic cytoplasm, and hyperchromatic nuclei (HE, ×400) (**A**), Chromogranin: strong cytoplasmic positivity in tumor cells (**B**), strong immunoreactivity (40%) for p53 (**C**), and a Ki-67 labeling index of 14% (**D**).

**Table 1:** Baseline patient and tumor characteristics (n = 111).

Variable	Value
Sex: Male/Female	51 (45.9%)/60 (54.1%)
Age at initial Dx—median (IQR), years	47 (34–55); range 9–75
Age at PC Dx—median (IQR), years	54 (40–61); range 9–80
Lineage: Corticotroph	53 (47.7%)
Lineage: Lactotroph	33 (29.7%)
Lineage: Null cell	13 (11.7%)
Lineage: Somatotroph	6 (5.4%)
Lineage: Other (thyrotroph, gonadotroph, plurihormonal)	6 (5.4%)
Functional tumor	85 (76.6%)
Time to PC Dx—median (IQR), months	72 (43–132); range 0–516
OS from PC Dx—median (IQR), months	19 (9.5–46); range 0–252
Status at last FU: Alive/Dead	50 (46.3%)/58 (53.7%)
Craniospinal metastases	68 (61.3%)
Systemic metastases	62 (55.9%)
Early metastasis (time to PC ≤ 24 months)	19 (17.1%)
Long survivor (OS from PC > 24 months)	39 (35.1%)
Ki-67 at initial Dx—median (IQR), %	7 (3–16)
Ki-67 at PC Dx—median (IQR), %	12 (4–24)

IQR, interquartile range; OS, overall survival; PC, pituitary carcinoma; Dx, diagnosis; FU, follow-up.

**Table 2:** Kaplan–Meier survival analysis by subgroup.

Stratification	Group	n	Median OS (Months)	95% CI	Events	*p* (Log-Rank)
Multimodal treatment	Single	32	12	12–48	22	
	Multimodal	66	45	24–NR	36	0.004
Any response	No response	67	21	12–30	48	
	Responder (CR/PR)	22	NR	60–NR	6	<0.001
Lineage	Non-corticotroph	51	24	19–48	31	
	Corticotroph	48	45	17–NR	27	0.330
Ki-67 initial Dx	<3%	9	156	17–NR	5	
	3–9%	13	32	12–NR	7	0.023 *
	≥10%	23	12	6–25	19	
Early metastasis	Late >24 month	80	20	—	—	
	Early ≤24 month	19	12	—	—	0.276
TMZ response	Non-responder	23	24	—	—	
	Responder	11	38	—	—	0.150 *

NR, not reached; CR, complete response; PR, partial response; OS, overall survival; TMZ, temozolomide. * Three-group log-rank (df = 2); n = 45 with available Ki-67 data; Mann–Whitney U *p*-value.

**Table 3:** Treatment utilization and response at PC stage (n = 111).

Treatment	n (%)	CR	PR	SD	PD	ORR	n Evaluable
Surgery	64 (57.7%)	—	—	—	—	—	—
Radiotherapy	66 (59.5%)	—	—	—	—	—	—
Temozolomide	42 (37.8%)	5	9	7	19	35%	40
ICI	8 (7.2%)	0	4	1	1	67%	6
Bevacizumab	8 (7.2%)	1	0	3	3	14%	7
PRRT	3 (2.7%)	0	0	0	2	0%	2
Multimodal (≥2 modalities)	73 (65.8%)	—	—	—	—	—	—

CR, complete response; PR, partial response; SD, stable disease; PD, progressive disease; ORR, objective response rate (CR + PR); ICI, immune checkpoint inhibitor; PRRT, peptide receptor radionuclide therapy.

**Table 4:** Univariate and multivariable Cox proportional hazards regression—predictors of OS (n = 99 evaluable).

Covariate	n	Events	HR (Univ.)	95% CI	*p* (Univ.)	HR (Adj.)	*p* (Adj.)
Age at PC Dx (per year)	98	58	1.024	1.006–1.042	0.009	1.018	0.049
Functional status (yes vs. no)	99	58	1.636	0.840–3.187	0.148	2.041	0.045
Multimodal treatment (yes vs. no)	98	58	0.466	0.270–0.802	0.006	0.678	0.189
Any treatment response (yes vs. no)	89	54	0.256	0.109–0.601	0.002	0.245	0.002
Sex (female vs. male)	99	58	1.169	0.690–1.980	0.562	—	—
Lineage: corticotroph (vs. other)	99	58	0.775	0.459–1.311	0.342	—	—
Early metastasis (yes vs. no)	99	58	1.412	0.759–2.626	0.276	—	—
Number of metastatic sites	97	57	0.990	0.808–1.213	0.923	—	—

HR, hazard ratio; CI, confidence interval; univ., univariate; adj., adjusted (multivariable). “—” = not included in multivariable model (univariate *p* ≥ 0.20). Multivariable model: n = 88; concordance index = 0.725; LR χ^2^ = 25.874, *p* < 0.001. INVASIVE_INITIAL excluded (constant in OS-evaluable subset). Analyses performed using Cox partial likelihood regression (Python 3.12/scipy).

**Table 5:** Binary logistic regression—independent predictors of long-survivor status (OS > 24 months; n = 87).

Predictor	OR	95% CI	*p*	Interpretation
Multimodal treatment (yes vs. no)	5.285	1.415–19.7	0.009	Independent predictor
Any treatment response (yes vs. no)	5.470	1.621–18.5	0.006	Independent predictor
Early metastasis ≤ 24 month (yes vs. no)	0.182	0.041–0.808	0.024	Reduced odds
Age at PC Dx (per year)	0.967	0.931–1.001	0.060	Borderline trend
Sex (female vs. male)	0.791	0.259–2.408	0.680	Not significant
Corticotroph lineage (yes vs. no)	1.556	0.548–4.418	0.407	Not significant

OR, odds ratio; CI, confidence interval; OS, overall survival; PC, pituitary carcinoma. Model fit: Nagelkerke R^2^ = 0.390; AUC = 0.818; accuracy 69.0%; sensitivity 57.1%; specificity 76.9%. Analysis performed in JASP 0.19.

**Table 6:** Summary of additional analyses.

Analysis	Test	n	Statistics	95% CI	*p*-Value
Ki-67, initial vs. PC diagnosis (paired)	Wilcoxon signed-rank	38	Median 7% → 12%	—	<0.001
Any treatment response vs. long-survivor status	Fisher’s exact	88	OR 6.00	1.89–21.6	<0.001
Multimodal treatment vs. long-survivor status	Fisher’s exact	98	OR 5.27	1.71–19.7	0.002
TMZ response vs. corticotroph lineage	Fisher’s exact	40	OR 0.47	0.13–1.76	0.327
Age at PC diagnosis vs. OS	Spearman correlation	98	ρ = −0.229	—	0.023
ROB category (JBI) vs. OS	Kruskal–Wallis	62 (of 66 with ROB scored)	Low 30.3%/Moderate 63.6%/High 6.1%	—	0.576

OS, overall survival; PC, pituitary carcinoma; TMZ, temozolomide; OR, odds ratio; CI, confidence interval; JBI, Joanna Briggs Institute; ROB, risk of bias. Odds ratios for Fisher’s exact comparisons are conditional maximum-likelihood estimates. ROB category defined as low concern (JBI score 4–5), moderate concern (score 3), or high concern (score ≤ 2); n = 66 reflects all ROB-scored cases, while the Kruskal–Wallis test (n = 62) is restricted to the subset with available OS data.

## Data Availability

The original contributions presented in this study are included in the article. Further inquiries can be directed to the corresponding author.

## References

[1] Dekkers O.M., Karavitaki N., Pereira A.M. (2020). The epidemiology of aggressive pituitary tumors (and its challenges). Rev. Endocr. Metab. Disord..

[2] Raverot G., Burman P., McCormack A., Heaney A., Petersenn S., Popovic V., Trouillas J., Dekkers O.M., European Society of Endocrinology (2018). European Society of Endocrinology Clinical Practice Guidelines for the management of aggressive pituitary tumours and carcinomas. Eur. J. Endocrinol..

[3] Trouillas J., Jaffrain-Rea M.-L., Vasiljevic A., Dekkers O., Popovic V., Wierinckx A., McCormack A., Petersenn S., Burman P., Raverot G. (2020). Are aggressive pituitary tumors and carcinomas two sides of the same coin? Pathologists reply to clinician’s questions. Rev. Endocr. Metab. Disord..

[4] Trouillas J., Burman P., McCormack A., Petersenn S., Popovic V., Dekkers O., Raverot G. (2018). Aggressive pituitary tumours and carcinomas: Two sides of the same coin?. Eur. J. Endocrinol..

[5] Casar-Borota O., Burman P., Lopes M.B. (2025). The 2022 WHO classification of tumors of the pituitary gland: An update on aggressive and metastatic pituitary neuroendocrine tumors. Brain Pathol..

[6] Hardy J., Vezina J.L. (1976). Transsphenoidal neurosurgery of intracranial neoplasm. Adv. Neurol..

[7] Wilson C.B. (1997). Surgical management of pituitary tumors. J. Clin. Endocrinol. Metab..

[8] Eisenhauer E.A., Therasse P., Bogaerts J., Schwartz L.H., Sargent D., Ford R., Dancey J., Arbuck S., Gwyther S., Mooney M. (2009). New response evaluation criteria in solid tumours: Revised RECIST guideline (version 1.1). Eur. J. Cancer.

[9] U.S. Department of Health and Human Services, National Institutes of Health, National Cancer Institute (2017). Common Terminology Criteria for Adverse Events (CTCAE) Version 5.0.

[10] Moola S., Munn Z., Sears K., Sfetcu R., Currie M., Lisy K., Tufanaru C., Qureshi R., Mattis P., Mu P. (2015). Conducting systematic reviews of association (etiology): The Joanna Briggs Institute’s approach. Int. J. Evid. Based Healthc..

[11] Nagaoka T., Takagawa K., Yokoyama K., Takamura T. (2026). A Case of Thyrotropin-Secreting Pituitary Carcinoma With Bone Metastases. JCEM Case Rep..

[12] Buchalska B., Kunicki J. (2026). Aggressive Pituitary Tumors and Pituitary Carcinomas: A Narrative Review of Clinical Features and Management. Neuroendocrinology.

[13] Raverot G., Burman P., Abreu A.P., Heaney A.P., van Hulsteijn L., Lin A.L., Marcus H., McCormack A., Minniti G., Petersenn S. (2025). Revised European Society of Endocrinology Clinical Practice Guideline for the management of aggressive pituitary tumours and pituitary carcinomas. Eur. J. Endocrinol..

[14] Christensen K., Mrvoljak M., Njoku C.L., Rajan S.S., Evbuomwan M.O., Smith M.C., Njoku C. (2025). Transformation of Lactotroph Pituitary Adenoma to Metastatic Pituitary Carcinoma: A Case Report. Cureus.

[15] Bioletto F., Bogeat C., Barat M., Benanteur N., Guignat L., Hage M., Garcia C., Calugaru V., Jacob J., Arrondeau J. (2025). Progression of potentially aggressive pituitary neuroendocrine tumors after radiotherapy: Risk factors, management, and outcomes. Eur. J. Endocrinol..

[16] Astafieva L.I., Chernov I., Kobyakov G., Trunin Y., Shishkina L., Shkarubo A., Fomichev D., Sidneva Y., Vagapova G., Kalinin P. (2025). Prolactin-secreting pituitary carcinomas with intra- and extracranial metastasis: Case report and review. Zhurnal Vopr. Neirokhirurgii Im. NN Burdenko.

[17] Yang Y., Liang W., Fan K., Yang T., Cheng J. (2024). Clinical features of pituitary carcinoma: Analysis based on a case report and literature review. Front. Endocrinol..

[18] Liu A., Van Uum S., Lee D., Hammond R.R., Ezzat S., Clemens K.K. (2024). A Rare Prolactin-secreting Pituitary Carcinoma With Epidural and Thecal Metastases. JCEM Case Rep..

[19] Esquivel J.E., Santos A.B., Hong A., Guillén A.C. (2024). Adrenocorticotropic Hormone Producing Pituitary Carcinoma in the Falx Cerebri, Retroclival Region, Ethmoidal Cells, and Other Locations. Cureus.

[20] DE Alcubierre D., Carretti A.L., Ducray F., Jouanneau E., Raverot G., Ilie M.D. (2024). Aggressive pituitary tumors and carcinomas: Medical treatment beyond temozolomide. Minerva Endocrinol..

[21] Raymond P., Raverot G., Ilie M.D. (2023). Outcome and prognostic factors for pituitary carcinomas: Lessons from a systematic review. Endocr.-Relat. Cancer.

[22] Messaoudi N., Assarrar I., Rouf S., Sefiani S., Latrech H. (2023). Non-functioning pituitary carcinoma: Case report and literature review. Int. J. Surg. Case Rep..

[23] Lamas C., Cámara R., Fajardo C., Remon-Ruiz P., Biagetti B., Guerrero-Pérez F., Araujo-Castro M., Mora M., Hanzu F., Iglesias P. (2023). Efficacy and safety of temozolomide in the treatment of aggressive pituitary neuroendocrine tumours in Spain. Front. Endocrinol..

[24] Derwich A., Sykutera M., Bromińska B., Rubiś B., Ruchała M., Sawicka-Gutaj N. (2023). The Role of Activation of PI3K/AKT/mTOR and RAF/MEK/ERK Pathways in Aggressive Pituitary Adenomas-New Potential Therapeutic Approach-A Systematic Review. Int. J. Mol. Sci..

[25] Wallace N.J., Devaiah A.K. (2022). Pituitary Carcinoma Diagnosis and Survival Improvement, with Affordable Care Act Correlation: A SEER Database Study. J. Neurol. Surg. B Skull Base.

[26] Van Mieghem E., Intan-Goey V., Buffet W., Lammens M., Van Loo P., Abrams P. (2022). Corticotroph pituitary carcinoma with skeletal metastases masquerading as ectopic ACTH syndrome: A long and winding road to diagnosis. Endocrinol. Diabetes Metab. Case Rep..

[27] Stelmachowska-Banaś M., Maksymowicz M., Kolasińska-Ćwikła A., Zieliński G., Korbonits M., Zgliczyński W. (2022). Pituitary carcinoma as a rare cause of liver metastases successfully treated with temozolomide. Pol. Arch. Intern. Med..

[28] Roelandt-Schumann F.E., Vergeer R.A., Korsten-Meijer A.G., Kramer M.C., Westerlaan H.E., Pott J.W., Nuver J., van den Berg G., den Dunnen W.F. (2022). An aggressive lactotroph pituitary tumor in a young male: A pituitary carcinoma without metastasis. Clin. Neuropathol..

[29] Raghu A.L.B., Vergeer R.A., Korsten-Meijer A.G., Kramer M.C., Westerlaan H.E., Pott J.W., Nuver J., Berg G.v.D., Dunnen W.F.D. (2022). Delayed Craniospinal Metastasis of Aggressive Nonfunctioning Pituitary Adenomas as Pituitary Carcinomas. J. Neurol. Surg. Part B Skull Base.

[30] Du P., Wu X., Lv K., Xiong J., Geng D. (2022). A Pituitary Carcinoma Patient With Cerebrospinal Fluid Dissemination Showing a Good Response to Temozolomide Combined With Whole-Brain and Spinal Cord Radiotherapy: A Case Report and Literature Review. Front. Oncol..

[31] Demir M.K., Yapıcıer O., Oral A., Yilmaz B., Kilic T. (2022). Non-functional recurrent pituitary adenoma with intracranial metastasis-Pituitary carcinoma: A case report and review of the literature. Neurochirurgie.

[32] Burman P., Trouillas J., Losa M., McCormack A., Petersenn S., Popovic V., Theodoropoulou M., Raverot G., Dekkers O.M., Guenego A. (2022). Aggressive pituitary tumours and carcinomas, characteristics and management of 171 patients. Eur. J. Endocrinol..

[33] Vekaria S., Chen F., Zan E., William C., Sen C., Lebowitz R., Zagzag D., Warren F.A., Brandler T.C., Agrawal N. (2021). Growth hormone secreting pituitary carcinomas: Case report and review of literature. Growth Horm. IGF Res..

[34] Remón-Ruiz P., Venegas-Moreno E., Dios-Fuentes E., Moreno J.M.C., Peña I.F., Garcia M.A., Japón-Rodriguez M.A., Roldán F., Fajardo E., Kaen A. (2021). A Silent Corticotroph Pituitary Carcinoma: Lessons From an Exceptional Case Report. Front. Endocrinol..

[35] Raverot G., Ilie M.D., Lasolle H., Amodru V., Trouillas J., Castinetti F., Brue T. (2021). Aggressive pituitary tumours and pituitary carcinomas. Nat. Rev. Endocrinol..

[36] Mori M., Frugoli A., Shah U., Barrows B., Westhoff T., Westra D. (2021). Recurrent Macroprolactinoma with Malignant Conversion to Carcinoma with Spinal Metastasis. Case Rep. Endocrinol..

[37] Kaya G., Turan B.S., Dagdelen S., Berker M., Tuncel M. (2021). 68Ga-DOTATATE PET/CT in Pituitary Carcinoma. Clin. Nucl. Med..

[38] Dai C., Sun B., Guan S., Wang W., Liu H., Li Y., Zhang J., Kang J. (2021). Evolution of a refractory prolactin-secreting pituitary adenoma into a pituitary carcinoma: Report of a challenging case and literature review. BMC Endocr. Disord..

[39] Dai C., Liang S., Sun B., Li Y., Kang J. (2021). Anti-VEGF Therapy in Refractory Pituitary Adenomas and Pituitary Carcinomas: A Review. Front. Oncol..

[40] Casar-Borota O., Boldt H.B., Engström B.E., Andersen M.S., Baussart B., Bengtsson D., Berinder K., Ekman B., Feldt-Rasmussen U., Höybye C. (2021). Corticotroph Aggressive Pituitary Tumors and Carcinomas Frequently Harbor ATRX Mutations. J. Clin. Endocrinol. Metab..

[41] Xu L., Khaddour K., Chen J., Rich K.M., Perrin R.J., Campian J.L. (2020). Pituitary carcinoma: Two case reports and review of literature. World J. Clin. Oncol..

[42] Todeschini A.B., Beer-Furlan A., Montaser A.S., Jamshidi A.O., Ghalib L., Chavez J.A., Lehman N.L., Prevedello D.M. (2020). Pituitary carcinomas: Review of the current literature and report of atypical case. Br. J. Neurosurg..

[43] Soon W.C., Czyz M., Dhir J. (2020). Metastatic Pituitary Carcinoma Causing Cord Compression. World Neurosurg..

[44] Rak B., Maksymowicz M., Grzywa T.M., Sajjad E., Pękul M., Włodarski P., Zieliński G. (2020). Pituitary tumours—A large retrospective single-centre study of over 2300 cases. Experience of a tertiary reference centre. Endokrynol. Pol..

[45] Carey R.M., Kuan E.C., Workman A.D., Patel N.N., Kohanski M.A., Tong C.C., Chen J., Palmer J.N., Adappa N.D., Brant J.A. (2020). A Population-Level Analysis of Pituitary Carcinoma from the National Cancer Database. J. Neurol. Surg. B Skull Base.

[46] Buchfelder M., Schlaffer S.M. (2020). Surgical treatment of aggressive pituitary adenomas and pituitary carcinomas. Rev. Endocr. Metab. Disord..

[47] Santos-Pinheiro F., Penas-Prado M., Kamiya-Matsuoka C., Waguespack S.G., Mahajan A., Brown P.D., Shah K.B., Fuller G.N., E McCutcheon I. (2019). Treatment and long-term outcomes in pituitary carcinoma: A cohort study. Eur. J. Endocrinol..

[48] Alshaikh O.M., Asa S.L., Mete O., Ezzat S. (2019). An Institutional Experience of Tumor Progression to Pituitary Carcinoma in a 15-Year Cohort of 1055 Consecutive Pituitary Neuroendocrine Tumors. Endocr. Pathol..

[49] Yoo F., Kuan E.C., Heaney A.P., Bergsneider M., Wang M.B. (2018). Corticotrophic pituitary carcinoma with cervical metastases: Case series and literature review. Pituitary.

[50] Lin A.L., Jonsson P., Tabar V., Yang T.J., Cuaron J., Beal K., Cohen M., Postow M., Rosenblum M., Shia J. (2018). Marked Response of a Hypermutated ACTH-Secreting Pituitary Carcinoma to Ipilimumab and Nivolumab. J. Clin. Endocrinol. Metab..

[51] Lenders N., McCormack A. (2018). Malignant transformation in non-functioning pituitary adenomas (pituitary carcinoma). Pituitary.

[52] Endo T., Watanabe M., Tominaga T., Ogawa Y. (2018). A Case of Pituitary Carcinoma Initially Diagnosed as an Ectopic Growth Hormone Producing Pituitary Adenoma with a High Ki-67 Labeling Index. J. Neurol. Surg. A Cent. Eur. Neurosurg..

[53] Bettencourt-Silva R., Pereira J., Belo S., Magalhães D., Queirós J., Carvalho D. (2018). Prolactin-Producing Pituitary Carcinoma, Hypopituitarism, and Graves’ Disease-Report of a Challenging Case and Literature Review. Front. Endocrinol..

[54] van der Vlist A., Snijders T., E Stades A.M., Spliet W.G.M., De Vos F.Y.F.L. (2017). Successful treatment of leptomeningeally metastasised pituitary carcinoma with temozolomide. Neth. J. Med..

[55] Touma W., Hoostal S., Peterson R.A., Wiernik A., SantaCruz K.S., Lou E. (2017). Successful treatment of pituitary carcinoma with concurrent radiation, temozolomide, and bevacizumab after resection. J. Clin. Neurosci..

[56] Krueger E.M., Seibly J. (2017). Seeding of a Pituitary Adenoma or Atypical Pituitary Carcinoma?. Cureus.

[57] Joehlin-Price A.S., Hardesty D.A., Arnold C.A., Kirschner L.S., Prevedello D.M., Lehman N.L. (2017). Case report: ACTH-secreting pituitary carcinoma metastatic to the liver in a patient with a history of atypical pituitary adenoma and Cushing’s disease. Diagn. Pathol..

[58] Chandler C.M., Lin X. (2017). Cytomorphology of metastatic pituitary carcinoma to the bone. Diagn. Cytopathol..

[59] Bengtsson D., Joost P., Aravidis C., Stenmark M.A., Backman A.-S., Melin B., von Salomé J., Zagoras T., Gebre-Medhin S., Burman P. (2017). Corticotroph Pituitary Carcinoma in a Patient With Lynch Syndrome (LS) and Pituitary Tumors in a Nationwide LS Cohort. J. Clin. Endocrinol. Metab..

[60] Yang Z., Zhang T., Gao H. (2016). Genetic aspects of pituitary carcinoma: A systematic review. Medicine.

[61] Seltzer J., Carmichael J.D., Commins D., Liu C.S., Omura E., Chang E., Zada G. (2016). Prolactin-Secreting Pituitary Carcinoma with Dural Metastasis: Diagnosis, Treatment, and Future Directions. World Neurosurg..

[62] Kamiya-Matsuoka C., Cachia D., Waguespack S.G., Crane C.H., Mahajan A., Brown P.D., Nam J.Y., McCutcheon I.E., Penas-Prado M. (2016). Radiotherapy with concurrent temozolomide for the management of extraneural metastases in pituitary carcinoma. Pituitary.

[63] Asa S.L., Ezzat S. (2016). Aggressive Pituitary Tumors or Localized Pituitary Carcinomas: Defining Pituitary Tumors. Expert. Rev. Endocrinol. Metab..

[64] Wei Z., Zhou C., Liu M., Yao Y., Sun J., Xiao J., Ma W., Zhu H., Wang R. (2015). MicroRNA involvement in a metastatic non-functioning pituitary carcinoma. Pituitary.

[65] Wang Y.Q., Fan T., Zhao X.G., Liang C., Qi X.L., Li J.Y. (2015). Pituitary carcinoma with intraspinal metastasis: Report of two cases and review of the literature. Int. J. Clin. Exp. Pathol..

[66] Sav A., Rotondo F., Syro L.V., Di Ieva A., Cusimano M.D., Kovacs K. (2015). Invasive, atypical and aggressive pituitary adenomas and carcinomas. Endocrinol. Metab. Clin. N. Am..

[67] Borba C.G., Batista R.L., Musolino N.R.d.C., Machado V.C., Alcantara A.E.E., da Silva G.O., Cescato V.A.S., Neto M.B.C.d.C. (2015). Progression of an Invasive ACTH Pituitary Macroadenoma with Cushing’s Disease to Pituitary Carcinoma. Case Rep. Oncol. Med..

[68] Zhong C., Yin S., Zhou P., Jiang S. (2014). Pituitary atypical adenoma or carcinoma sensitive to temozolomide combined with radiation therapy: A case report of early identification and management. Turk. Neurosurg..

[69] Park K.S., Hwang J.-H., Hwang S.-K., Kim S., Park S.-H. (2014). Pituitary carcinoma with fourth ventricle metastasis: Treatment by excision and Gamma-knife radiosurgery. Pituitary.

[70] Mendola M., Passeri E., Ambrosi B., Corbetta S. (2014). Multiple cerebral hemorrhagic foci from metastases during temozolomide treatment in a patient with corticotroph pituitary carcinoma. J. Clin. Endocrinol. Metab..

[71] Lee H.H., Hung S.-H., Tseng T.-M., Lin Y.-H., Cheng J.-C. (2014). Undifferentiated carcinoma of the pituitary gland: A case report and review of the literature. Oncol. Lett..

[72] Hirohata T., Ishii Y., Matsuno A. (2014). Treatment of pituitary carcinomas and atypical pituitary adenomas: A review. Neurol. Med. Chir..

[73] Hansen T.M., Batra S., Lim M., Gallia G.L., Burger P.C., Salvatori R., Wand G., Quinones-Hinojosa A., Kleinberg L., Redmond K.J. (2014). Invasive adenoma and pituitary carcinoma: A SEER database analysis. Neurosurg. Rev..

[74] Zhou Q., Chang H., Gao Y., Cui L. (2013). Tumor-to-tumor metastasis from pituitary carcinoma to radiation-induced meningioma. Neuropathology.

[75] Vieira Neto L., Chimelli L., Pereira P.J.d.M., Gasparetto E.L., Bines J., Wildemberg L.E.A., Gadelha M.R. (2013). The role of temozolomide in the treatment of a patient with a pure silent pituitary somatotroph carcinoma. Endocr. Pr..

[76] Tanaka T., Kato N., Aoki K., Watanabe M., Arai T., Hasegawa Y., Abe T. (2013). Long-term follow-up of growth hormone-producing pituitary carcinoma with multiple spinal metastases following multiple surgeries: Case report. Neurol. Med. Chir..

[77] Shastri B.R., Nanda A., Fowler M., Levine S.N. (2013). Adrenocorticotropic hormone-producing pituitary carcinoma with intracranial metastases. World Neurosurg..

[78] Lall R.R., Shafizadeh S.F., Lee K.H., Mao Q., Mehta M., Raizer J., Bendok B.R., Chandler J.P. (2013). Orbital metastasis of pituitary growth hormone secreting carcinoma causing lateral gaze palsy. Surg. Neurol. Int..

[79] Kovács G.L., Góth M., Rotondo F., Scheithauer B.W., Carlsen E., Saadia A., Hubina E., Kovács L., Szabolcs I., Nagy P. (2013). ACTH-secreting Crooke cell carcinoma of the pituitary. Eur. J. Clin. Investig..

[80] Cornell R.F., Kelly D.F., Bordo G., Carroll T.B., Duong H.T., Kim J., Takasumi Y., Thomas J.P., Wong Y.L., Findling J.W. (2013). Chemotherapy-induced regression of an adrenocorticotropin-secreting pituitary carcinoma accompanied by secondary adrenal insufficiency. Case Rep. Endocrinol..

[81] Philippon M., Morange I., Barrie M., Barlier A., Taieb D., Dufour H., Conte-Devolx B., Brue T., Castinetti F. (2012). Long-term control of a MEN1 prolactin secreting pituitary carcinoma after temozolomide treatment. Ann. Endocrinol..

[82] Oh M.C., Tihan T., Kunwar S., Blevins L., Aghi M.K. (2012). Clinical management of pituitary carcinomas. Neurosurg. Clin. N. Am..

[83] Morokuma H., Ando T., Hayashida T., Horie I., Inoshita N., Murata F., Ueki I., Nakamura K., Imaizumi M., Usa T. (2012). A case of nonfunctioning pituitary carcinoma that responded to temozolomide treatment. Case Rep. Endocrinol..

[84] Arnold P.M., Ratnasingam D., O’Neil M.F., Johnson P.L. (2012). Pituitary carcinoma recurrent to the lumbar intradural extramedullary space: Case report. J. Spinal Cord. Med..

[85] Salehi F., Scheithauer B.W., Kros J.M., Lau Q., Fealey M., Erickson D., Kovacs K., Horvath E., Lloyd R.V. (2011). MGMT promoter methylation and immunoexpression in aggressive pituitary adenomas and carcinomas. J. Neurooncol..

[86] Ono M., Miki N., Amano K., Hayashi M., Kawamata T., Seki T., Takano K., Katagiri S., Yamamoto M., Nishikawa T. (2011). A case of corticotroph carcinoma that caused multiple cranial nerve palsies, destructive petrosal bone invasion, and liver metastasis. Endocr. Pathol..

[87] Dudziak K., Honegger J., Bornemann A., Horger M., Müssig K. (2011). Pituitary carcinoma with malignant growth from first presentation and fulminant clinical course--case report and review of the literature. J. Clin. Endocrinol. Metab..

[88] Sansur C.A., Oldfield E.H. (2010). Pituitary carcinoma. Semin. Oncol..

[89] Curtò L., Torre M.L., Ferraù F., Pitini V., Altavilla G., Granata F., Longo M., Hofland L.J., Trimarchi F., Cannavò S. (2010). Temozolomide-induced shrinkage of a pituitary carcinoma causing Cushing’s disease—Report of a case and literature review. Sci. World J..

[90] Hagen C., Schroeder H.D., Hansen S., Andersen M. (2009). Temozolomide treatment of a pituitary carcinoma and two pituitary macroadenomas resistant to conventional therapy. Eur. J. Endocrinol..

[91] Guzel A., Tatli M., Senturk S., Guzel E., Cayli S.R., Sav A. (2008). Pituitary carcinoma presenting with multiple metastases: Case report. J. Child. Neurol..

[92] Koyama J., Ikeda K., Shose Y., Kimura M., Obora Y., Kohmura E. (2007). Long-term survival with non-functioning pituitary carcinoma—Case report. Neurol. Med. Chir..

[93] Guastamacchia E., Triggiani V., Tafaro E., De Tommasi A., De Tommasi C., Luzzi S., Sabba’ C., Resta F., Terreni M.R., Losa M. (2007). Evolution of a prolactin-secreting pituitary microadenoma into a fatal carcinoma: A case report. Minerva Endocrinol..

[94] Choi G., Choi H.-J., Kim Y.M., Choi S.H., Cho Y.C., Suh J.H., Cha H.J. (2007). Pituitary carcinoma with mandibular metastasis: A case report. J. Korean Med. Sci..

[95] Tena-Suck M.L., Salinas-Lara C., Sánchez-García A., Rembao-Bojórquez D., Ortiz-Plata A. (2006). Late development of intraventricular papillary pituitary carcinoma after irradiation of prolactinoma. Surg. Neurol..

[96] Fadul C.E., Kominsky A.L., Meyer L.P., Kingman L.S., Kinlaw W.B., Rhodes C.H., Eskey C.J., Simmons N.E. (2006). Long-term response of pituitary carcinoma to temozolomide. Report of two cases. J. Neurosurg..

[97] Yamashita H., Nakagawa K., Tago M., Nakamura N., Shiraishi K., Yamauchi N., Ohtomo K. (2005). Pathological changes after radiotherapy for primary pituitary carcinoma: A case report. J. Neurooncol..

[98] Ayuk J., Natarajan G., Geh J.I., Mitchell R.D., Gittoes N.J.L. (2005). Pituitary carcinoma with a single metastasis causing cervical spinal cord compression. Case report. J. Neurosurg. Spine.

[99] Tysome J., Gnanalingham K.K., Chopra I., Mendoza N. (2004). Intradural metastatic spinal cord compression from ACTH-secreting pituitary carcinoma. Acta Neurochir..

[100] Noda M., Suzuki R., Moriya M., Itokawa H., Asai J.-I., Nagashima G., Fujimoto T. (2004). A case of prolactin-secreting pituitary carcinoma and its histological findings. Brain Tumor Pathol..

[101] Roncaroli F., Scheithauer B.W., Young W.F., Horvath E., Kovacs K., Kros J.M., Al-Sarraj S., Lloyd R.V., Faustini-Fustini M. (2003). Silent corticotroph carcinoma of the adenohypophysis: A report of five cases. Am. J. Surg. Pathol..

[102] Sironi M., Cenacchi G., Cozzi L., Tonnarelli G., Iacobellis M., Trere D., Assi A. (2002). Progression on metastatic neuroendocrine carcinoma from a recurrent prolactinoma: A case report. J. Clin. Pathol..

[103] Pichard C., Gerber S., Laloi M., Kujas M., Clemenceau S., Ponvert D., Bruckert E., Turpin G. (2002). Pituitary carcinoma: Report of an exceptional case and review of the literature. J. Endocrinol. Investig..

[104] Landman R.E., Horwith M., Peterson R.E., Khandji A.G., Wardlaw S.L. (2002). Long-term survival with ACTH-secreting carcinoma of the pituitary: A case report and review of the literature. J. Clin. Endocrinol. Metab..

[105] Zahedi A., Booth G.L., Smyth H.S., Farrell W.E., Clayton R.N., Asa S.L., Ezzat S. (2001). Distinct clonal composition of primary and metastatic adrencorticotrophic hormone-producing pituitary carcinoma. Clin. Endocrinol..

[106] Kumar K., Macaulay R.J., Kelly M., Pirlot T. (2001). Absent p53 immunohistochemical staining in a pituitary carcinoma. Can. J. Neurol. Sci..

[107] Holthouse D.J., Robbins P.D., Kahler R., Knuckey N., Pullan P. (2001). Corticotroph pituitary carcinoma: Case report and literature review. Endocr. Pathol..

[108] De Sousa S.M.C., McCormack A.I., Feingold K.R., Adler R.A., Ahmed S.F., Anawalt B., Blackman M.R., Chrousos G., Corpas E., de Herder W.W., Dhatariya K., Dungan K. (2000). Aggressive Pituitary Tumors and Pituitary Carcinomas. Endotext.

[109] Masuda T., Akasaka Y., Ishikawa Y., Ishii T., Isshiki I., Imafuku T., Ogihara T., Miyazaki H., Asuwa N. (1999). An ACTH-producing pituitary carcinoma developing Cushing’s disease. Pathol. Res. Pr..

[110] Kemink S.A., Wesseling P., Pieters G.F.F.M., Verhofstad A.A.J., Hermus A.R.M.M., Smals A.G.H. (1999). Progression of a Nelson’s adenoma to pituitary carcinoma; a case report and review of the literature. J. Endocrinol. Investig..

[111] Lormeau B., Miossec P., Sibony M., Valensi P., Attali J.-R. (1997). Adrenocorticotropin-producing pituitary carcinoma with liver metastasis. J. Endocrinol. Investig..

[112] Garrão A.F., Sobrinho L., Oliveira P., Bugalho M., Boavida J., Raposo J., Loureiro M., Limbert E., Costa I., Antunes J. (1997). ACTH-producing carcinoma of the pituitary with haematogenic metastases. Eur. J. Endocrinol..

[113] Saeger W., Lubke D. (1996). Pituitary Carcinomas. Endocr. Pathol..

[114] Gollard R., Kosty M., Cheney C., Copeland B., Bordin G. (1995). Prolactin-secreting pituitary carcinoma with implants in the cheek pouch and metastases to the ovaries. A case report and literature review. Cancer.

[115] Beauchesne P., Trouillas J., Barral F. (1995). Jacques Brunon Gonadotropic pituitary carcinoma: Case report. Neurosurgery.

[116] Cusimano M.D., Ohori P., Martinez A.J., Jungreis C., Wright D.C. (1994). Pituitary carcinoma. Skull Base Surg..

[117] Mixson A.J., Friedman T.C., Katz D.A., Feuerstein I.M., Taubenberger J.K., Colandrea J.M., Doppman J.L., Oldfield E.H., Weintraub B.D. (1993). Thyrotropin-secreting pituitary carcinoma. J. Clin. Endocrinol. Metab..

[118] Atienza D.M., Vigersky R.J., Lack E.E., Carriaga M., Rusnock E.J., Tsou E., Cerrone F., Kattah J.G., Sausville E.A. (1991). Prolactin-producing pituitary carcinoma with pulmonary metastases. Cancer.

[119] Gatti G., Limone P. (1984). ACTH-producing hypophyseal carcinoma monitored by computed tomography. Diagn. Imaging Clin. Med..

[120] Wilson D.F. (1982). Pituitary carcinoma occurring as middle ear tumor. Otolaryngol. Head. Neck Surg..

[121] McCormack A., Dekkers O.M., Petersenn S., Popovic V., Trouillas J., Raverot G., Burman P., Hubalewska-Dydejezky A., Assie G., Bach L. (2018). Treatment of aggressive pituitary tumours and carcinomas: Results of a European Society of Endocrinology (ESE) survey 2016. Eur. J. Endocrinol..

[122] Duhamel C., Ilie M.D., Salle H., Nassouri A.S., Gaillard S., Deluche E., Assaker R., Mortier L., Cortet C., Raverot G. (2020). Immunotherapy in Corticotroph and Lactotroph Aggressive Tumors and Carcinomas: Two Case Reports and a Review of the Literature. J. Pers. Med..

[123] Bordo G., Duong H., McLaughlin N., Cornell R., Findling J.W., Kelly D.F. (2012). Case report: Silent corticotroph adenoma progressing to cushing disease then to pituitary carcinoma with dramatic treatment response. Endocr. Rev..

[124] Farrell W.E., Coll A.P., Clayton R.N., Harris P.E. (2003). Corticotroph carcinoma presenting as a silent corticotroph adenoma. Pituitary.

[125] Raverot G., Dantony E., Beauvy J., Vasiljevic A., Mikolasek S., Borson-Chazot F., Jouanneau E., Roy P., Trouillas J. (2017). Risk of Recurrence in Pituitary Neuroendocrine Tumors: A Prospective Study Using a Five-Tiered Classification. J. Clin. Endocrinol. Metab..

